# Fabrication Flexible and Luminescent Nanofibrillated Cellulose Films with Modified SrAl_2_O_4_: Eu, Dy Phosphors via Nanoscale Silica and Aminosilane

**DOI:** 10.3390/nano8050352

**Published:** 2018-05-22

**Authors:** Longfei Zhang, Shaoyi Lyu, Zhilin Chen, Siqun Wang

**Affiliations:** 1 Research Institute of Wood Industry, Chinese Academy of Forestry, Beijing 100091, China; lzhang96@utk.edu (L.Z.); lvsy@caf.ac.cn (S.L.); 2 Center for Renewable Carbon, University of Tennessee, Knoxville, TN 37996, USA

**Keywords:** nanofibrillated cellulose, luminescent, phosphor, modification, composite film

## Abstract

Flexible 2,2,6,6-tetramethylpiperidine-1-oxyl radical (TEMPO)-oxidized nanofibrillated cellulose (ONFC) films with long afterglow luminescence containing modified SrAl_2_O_4_: Eu^2+^, Dy^3+^ (SAOED) phosphors were fabricated by a template method. Tetraethyl orthosilicate (TEOS) and (3-aminopropyl) trimethoxy-silane (APTMS) were employed cooperatively to improve the water resistance and compatibility of the SAOED particles in the ONFC suspension. The structure and morphology after modification evidenced the formation of a superior SiO_2_ layer and coarse amino-compounds on the surface of the phosphors. Homogeneous dispersions containing ONFC and the modified phosphors were prepared and the interface of composite films containing the amino-modified particles showed a more closely packed structure and had less voids at the interface between the cellulose and luminescent particles than that of silica-modified phosphors. The emission spectra for luminescent films showed a slight blue shift (3.2 nm) at around 512 nm. Such flexible films with good luminescence, thermal resistance, and mechanical properties can find applications in fields like luminous flexible equipment, night indication, and portable logo or labels.

## 1. Introduction

Nanofibrillated cellulose (NFC) is a natural biomass material with unique properties such as low density, high aspect ratio, high mechanical strength, high degree of crystal structure, and excellent biocompatibility and biodegradability [1,2]. Generally, it is prepared from wood or plant fibers using the acid [3], mechanical, or enzymatic method [4] and is expected to be one of the most promising alternative to petroleum-based polymers. In addition, because of the presence of a large number of hydroxyl groups [5], the cellulose nanofibers are liable to form a three-dimensional skeleton structure upon dehydration [6]. Owing to these properties, NFC has gained huge attention in the fabrication of functional composites for thermal insulation films [7], in flexible electronics [8], and in biomedicine engineering, etc.

Currently, many attempts have been made to combine cellulose fibers and luminescent materials for applications in optics [9,10,11], such as hydrogels [12], aerogels [13], and thin films [14,15]. These as-prepared luminescent films are widely used in optoelectronic devices, thin-film solar cells, display devices, and other fields. For instance, some rare-earth up-converting nanoparticles were prepared and assembled with NFC to fabricate a sheet of hybrid luminescent paper and a visible green light was observed under a 980 nm laser excitation [16]. However, the luminescent materials produced in these studies mainly involved aqueous dispersible luminescent particles and various quantum dots under high-energy laser excitation. Rare-earth-doped aluminate micro- and nanoparticles (SrAl_2_O_4_: Eu^2+^, Dy^3+^, denoted as SAOED) are environment-friendly functional inorganic materials. These SAOED particles show a high luminous efficiency and a long afterglow time, and thus are widely used in luminous displays [17], warning signs [18], and advertisement cards [19]. However, these particles are prone to hydrolysis in high-moisture environments and because of the damage of their crystal structures it leads to non-luminescence [20]. Upon hydrolysis, these particles become non-luminous, which seriously restricts their applications. Therefore, some researchers introduced water-free ionic liquids to prepare luminescent cellulose films in order to avoid the hydrolysis of phosphors [21]. However, this process is relatively difficult and the high-cost ionic liquids are not easy to recycle. So far, the cellulose nanofibers have been initially prepared in an aqueous system, it is of great significance to develop functional nanocellulose films in water solutions with relatively simple methods.

To incorporate the SAOED into the NFC aqueous soft matrix and obtain good mechanical properties of the composite films, the biggest challenge also includes that SAOED phosphors are difficult to disperse with organic cellulose and difficult to form a firm combination with cellulose due to their unavoidable hydrolysis, agglomeration, and low chemical activity. Therefore, it is necessary to modify SAOED phosphors to improve their water resistance and to control their surface compositions before using them in NFC hydrosol systems. Up to date, several physical and chemical strategies have been used to improve the hydrolytic resistance of phosphors [22,23,24]. The importance of silicone oil [25] and SiO_2_ coating layer [26,27] were confirmed in terms of enhancing the water resistance of phosphor particles. However, even though the water resistance of phosphors was significantly improved, these approaches did not address the problem of low surface activity of phosphors, especially in water-based systems containing cellulose. This could be confirmed from a study of using a mixture of several cross-linked components to enhance the interaction between the phosphor particles and cotton fibers [28]. To the best of our knowledge, except for a study reporting the direct introduction of SAOED phosphors into microcrystalline cellulose in an ionic liquid solution for casting films [29], there has been no report of amino-modification of these phosphor particles by SiO_2_@APTMS and their introduction into a nanocellulose matrix in an aqueous medium. Comparing with other nanocelluloses, TEMPO-oxidized nanofibrillated cellulose (ONFC) was prepared by a 2,2,6,6-tetramethylpiperidine-1-oxyl radical (TEMPO)-mediated oxidation method [30]. It is stable in homogeneous suspensions with rich surface carboxylation and has some additionally distinct characters [31], especially smaller size, higher surface chemical activity, stability of the suspension, and higher light transmittance. Hydrogen bonds and electrostatic attraction between the negatively charged carboxylate groups (–COO^−^) of ONFC and the positively charged ammonium groups (–NH_3_^+^) of the modified phosphors may be the driving forces for a better interface combination [31,32]. Therefore, these strong bonds also ensure a high compatibility between the ONFC and modified SAOED particles.

Herein, the aim of this research was to fabricate high-strength flexible films with long afterglow luminescence simultaneously based on nanoscale SiO_2_@APTMS modified SAOED phosphors as functional filler and ONFC as reinforcing skeleton in aqueous systems. Two types of surface modification comprising SiO_2_ and SiO_2_@APTMS were carried out to improve the water resistance and surface activity of the SAOED particles. Furthermore, the effect of different ratios of the SAOED and ONFC on the luminescence and mechanical properties of the resulting composite films were also investigated.

## 2. Materials and Methods

### 2.1. Materials

The ONFC suspension prepared from hardwood pulp using a TEMPO-oxidation method was purchased from Hao Jia Nanocellulose Technology Co., Ltd., Tianjin, China. The average diameter and length of the fibers was 10–20 nm and 0.8–2.0 μm, respectively. The carboxyl substitution degree of ONFC was 0.42 and the charge density was 1.5 meq/g. Tetraethyl orthosilicate (TEOS, 28%), oxalic acid dehydrate (C_2_H_2_O_4_·2H_2_O), and ethanol (C_2_H_5_OH) were analytical reagent (AR) and acquired from Sinopharm Chemical Reagent Co., Ltd., Beijing, China. (3-aminopropyl) trimethoxy-silane (APTMS, 97%) was purchased from Sigma-Aldrich (Shanghai, China). The SAOED phosphors were obtained from Lu Ming Technology Group Co., Ltd., Dalian, China.

### 2.2. Surface Modification of SAOED Phosphors

Two methods were used to modify the SAOED particles to obtain coated phosphors. The first method involved silica modification (using TEOS) and the second one was amination (using APTMS) of the SiO_2_-coated phosphors obtained from the first method. First, the SiO_2_ coating was prepared by a sol-gel route using TEOS as the precursor. Deionized water (4.2 mL) and 10 mL of TEOS were added into a three-necked flask. The molar ratio of TEOS to C_2_H_5_OH and water was theoretically calculated as 1:20:35. The pH value of the mixture was adjusted to 2.5 by C_2_H_2_O_4_·2H_2_O, and stirred for 45 min in a water bath at 60 °C to form a homogeneous sol. Then, the SAOED phosphors (2.0 g) were added to the flask, and the mixture was stirred at 60 °C until the phosphors swelled and transferred into gel-like precipitates. The precipitates were then calcined at 500 °C for 2 h under a N_2_ atmosphere to obtain the silica-modified phosphors denoted as SiO_2_@SAOED. Secondly, the SiO_2_@SAOED particles (1.0 g) were added into a flask with 50 g of C_2_H_5_OH and 178 μL of APTMS. The mixture was stirred for 8 h at room temperature. After that, the resulting mixture was then centrifuged, washed three times with aqueous ethanol solution, and dried at 70 °C for 4 h to obtain surface-aminated phosphors denoted as NH_2_@SiO_2_@SAOED. For comparison, SAOED phosphors only modified with APTMS (without pre-coated of SiO_2_ coatings) were denoted as NH_2_@SAOED.

### 2.3. Fabrication of Luminescent ONFC Composite Films

**Scheme 1:**
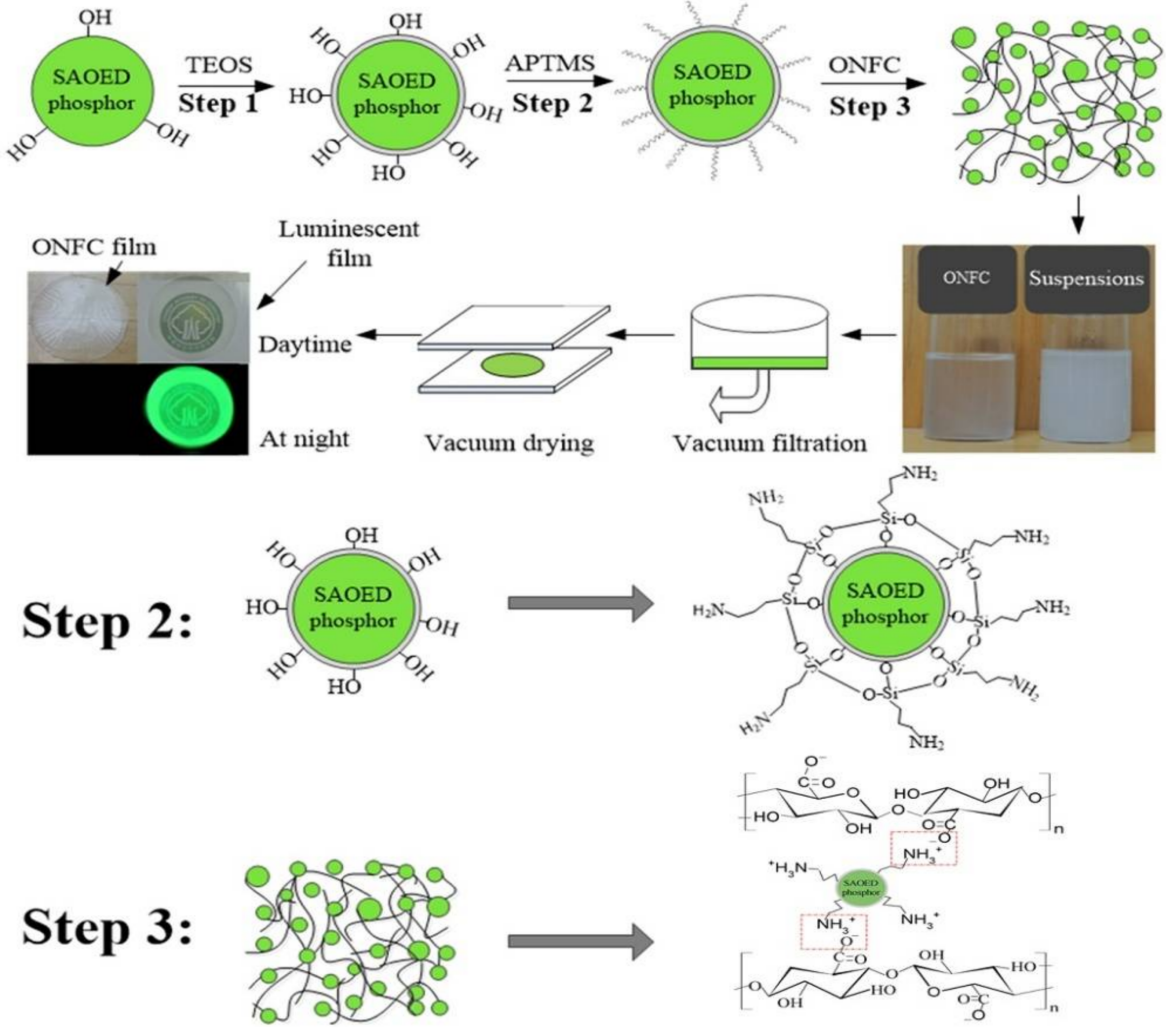
Schematic diagrams of preparing modified SrAl_2_O_4_: Eu^2+^, Dy^3+^ (SAOED) phosphor particles and synthesis procedure to obtain luminescent TEMPO-oxidized nanofibrillated cellulose (ONFC) composite films.

A certain amount of deionized water was added to the ONFC sol and ultrasonically dispersed for 30 min to form a 0.2 wt % suspension. Then, different wt % contents of modified phosphors were directly added into different beakers of the above ONFC dispersions (ONFC/phosphor = 7/3, 6/4, 5/5, 4/6, and 3/7). The different mass fractions were calculated according to the dry weight of ONFC. The dispersions were mixed with magnetic stirring, and an ultrasonic processing of 220 W for 30 min was needed to ensure homogeneous mixing between ONFC suspension and modified phosphors. The ONFC films with phosphors were then prepared using a template method [33,34]. As shown in Scheme 1, the dispersions were vacuum filtered by a membrane filter (PTFE type, pore size 100 nm) for 4–6 h to form a wet ONFC mat. Subsequently, the wet mat was carefully sandwiched between two parallel glass plates with a weight load of 800 g. Then, all devices including the fiber mats were dried at 40 °C overnight for 48 h. Finally, flat films with a diameter of 70 mm and a thickness of 30–40 μm were obtained. For comparison, a control ONFC film without phosphors was also fabricated using a similar method.

### 2.4. Characterization

The surface morphologies were characterized by scanning electron microscopy (SEM, Hitachi S-4800, Tokyo, Japan) at an accelerating voltage of 5.0 kV. Prior to SEM test, the phosphors and film specimens were adhered to a conductive tape and gold coated to eliminate electrostatic charging. The average thickness of the coating layer deposited onto phosphors was measured by transmission electron microscopy (TEM) (Tecnai G2, FEI Corp., Hillsboro, TX, USA) at an accelerating voltage of 120 kV. The diluted aqueous dispersion of different kinds of phosphors was added dropwise on a 200 mesh holey carbon film (grid of 1.2 µm), respectively. The particle size distributions of SAOED phosphors before and after surface coated modification were measured by Zetasizer Nano Series instrument (Nano ZS, Malvern Instruments Ltd., Malvern, UK). The X-ray photoelectron spectroscopy (XPS) spectra were recorded on a Thermo Escalab 250xi system (Thermo Electron Scientific Instruments Co. Ltd., Massachusetts, Waltham, MA, USA). Fourier transform infrared (FTIR) spectra were conducted over the wavelength range 4000–400 cm^−1^. The surface chemical composition was examined by energy dispersive spectroscopy (EDS, JEM-2100, JEOL, Ltd., Tokyo, Japan). The EDS-mappings of the film specimens were collected from an area of 750 μm × 750 μm with acquisition time of 240 s at 10 kV. Thermal stability was examined using a thermal gravimetric analyzer (TGA; DT-30, Shimadu Corp., Kyoto, Japan). All the specimens were heated from 40 to 700 °C at a heating rate of 10 °C/min under N_2_ atmosphere. The phase structures were examined by powder X-ray diffraction (XRD) analysis using the Cu-Kα radiation (λ = 1.5406Å). The continuous scanning rate (2θ ranging from 10 to 80°) was 5°/min. The Zeta potentials were determined using the same Zetasizer Nano series. All the test samples were diluted with deionized water and carried out at room temperature. The excitation and emission spectra were recorded using a spectrophotometer (Hitachi F-4500, Tokyo, Japan) equipped with a xenon lamp as the excitation source. The slit width was 2.5 nm, the excitation wavelength was 363 nm, and the scan speed was 20 nm/s. The same quality of phosphor samples was used for comparative analysis of the spectra intensity. The afterglow decay curves were obtained using the life-time mode in the fluorescence tester (excitation illumination: 1000 lx, excitation time: 15 min). The mechanical performance of ONFC and luminescent films, with a dimension of 5.0 cm × 0.5 cm and a thickness of 30–40 μm, was carried out with a tensile testing device (INSTRON-5582, Instron Corp., Boston, MA, USA). Prior to the test, all the films were processed in a temperature humidity chamber (25 °C and 65% RH) for more than 24 h. Each film was fixed with rubber sheets to ensure upper and lower clamps on a vertical line. The speed of the load head was 1 mm/min and five duplicate specimens were repeated for the final analysis. The presented values (for various parameters) in this study are the average values.

## 3. Results and discussions

### 3.1. Surface Modification of SAOED Phosphors

**Figure 1:**
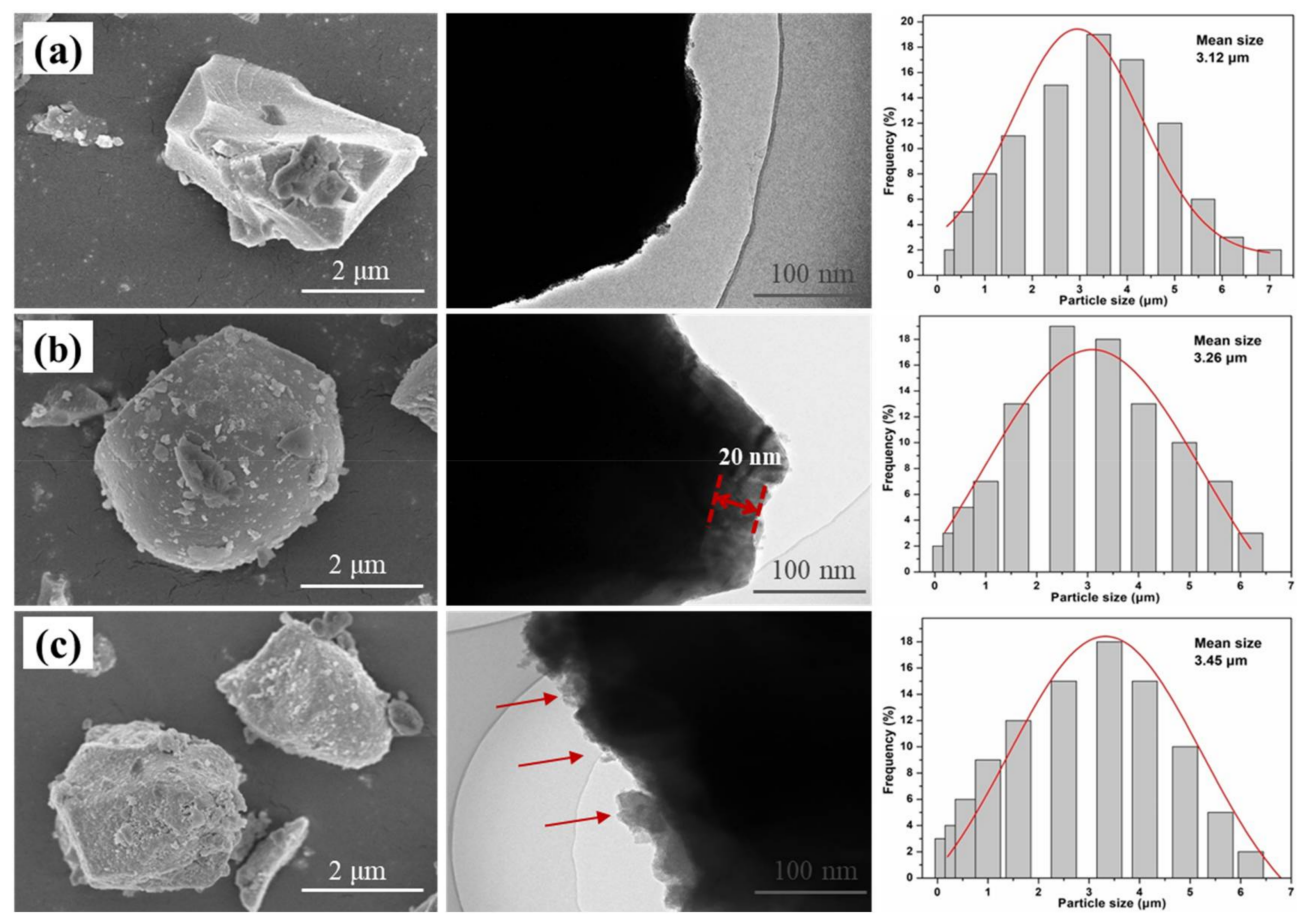
Scanning electron microscopy (SEM) micrographs, transmission electron microscopy (TEM) images and the distribution of average particle size of SAOED particles before and after modification: (**a**) uncoated SAOED phosphor; (**b**) SiO_2_ coated SAOED (SiO_2_@SAOED); (**c**) (3-aminopropyl) trimethoxy-silane (APTMS) coated SiO_2_@SAOED phosphors (NH_2_@SiO_2_@SAOED).

The SEM micrographs and TEM images of the SAOED particles before and after modification are shown in Figure 1 and Figure S1. Compared with the smooth surface of untreated phosphor (Figure 1a), the morphology of coated SAOED particles are obviously becoming coarse (Figure 1b,c). As shown in Figure 1b, a thin layer (approximately 20 nm thick) is clearly observed on the surface of SiO_2_-coated particles. For APTMS-coated SiO_2_@SAOED phosphors (Figure 1c), the surface morphology is a little rough comparing with the SiO_2_-coated phosphors (Figure 1b). It is likely that polymer segments of APTMS are grafted on the surface of SiO_2_@SAOED phosphors. The average particle size of phosphors increased slightly from 3.12 to 3.45 μm after nanoscale coating modification without negative effect on its overall distribution. However, further analyses (XPS, EDS, and FTIR) were required to confirm the deposition of APTMS on the SiO_2_@SAOED particles.

**Figure 2:**
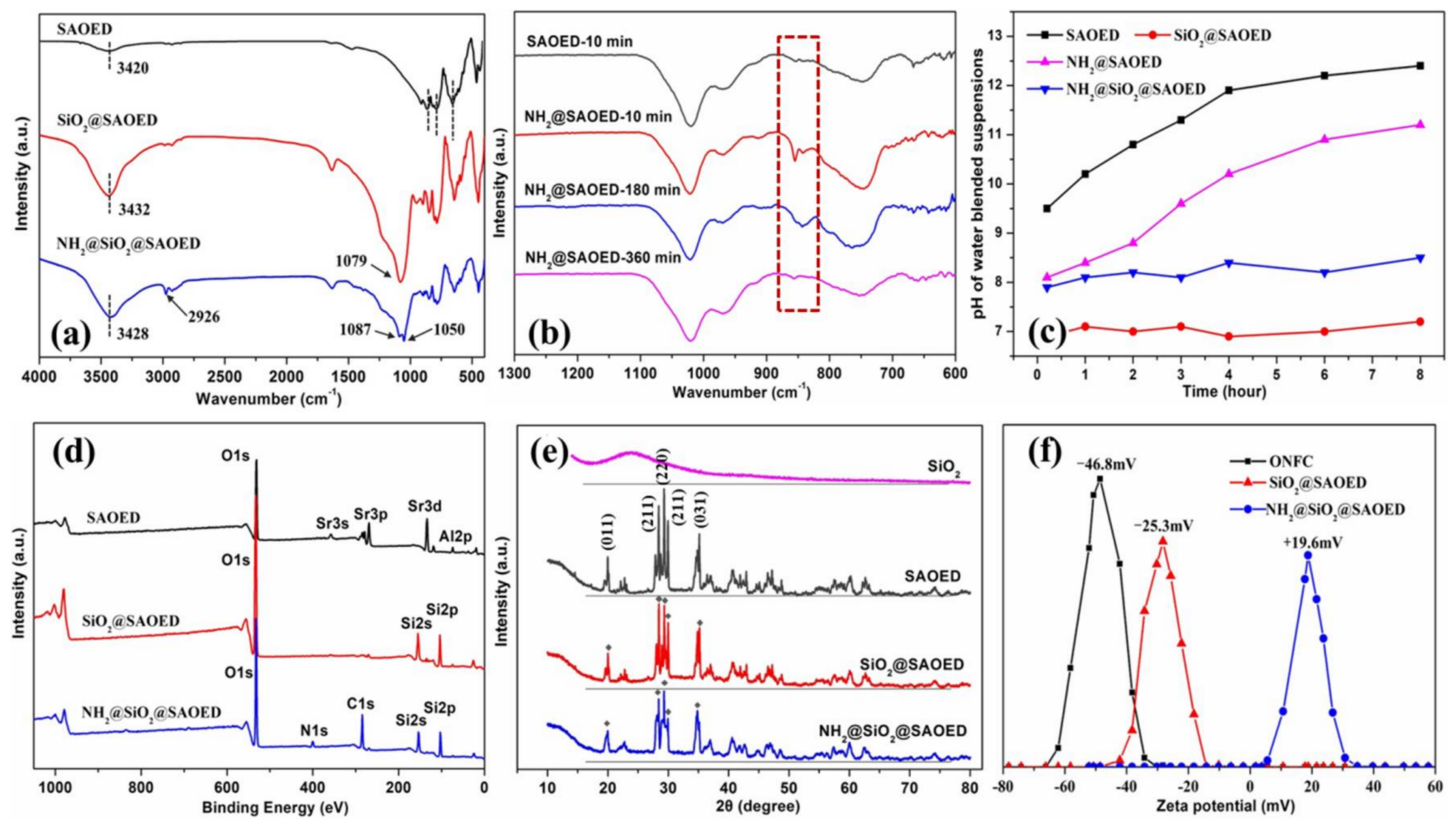
Chemical characteristics of SAOED phosphors before and after surface modification: (**a**) Fourier transform infrared (FTIR) spectra of SAOED, SiO_2_@SAOED, and NH_2_@SiO_2_@SAOED; (**b**) FTIR spectra of SAOED and pure APTMS-modified phosphors (NH_2_@SAOED) after immersion in water for different time; (**c**) pH values of different phosphor particle aqueous suspensions with different immersion time; (**d**) X-ray photoelectron spectroscopy (XPS) wide scan spectra of SAOED, SiO_2_@SAOED and NH_2_@SiO_2_@SAOED; (**e**) X-ray diffraction patterns for SAOED, SiO_2_@SAOED, NH_2_@SiO_2_@SAOED and control SiO_2_ particles; (**f**) Zeta potentials of modified particles comparing with ONFC in aqueous suspensions.

The FTIR spectra of the SiO_2_@SAOED, NH_2_@SiO_2_@SAOED, and uncoated SAOED phosphors are shown in Figure 2a. As shown in Figure 2a, the absorption bands peaked at 850, 770, and 642 cm^−1^ for SAOED are attributed to the stretching vibration modes of SrAl_2_O_4_ [27]. These corresponding characteristic peaks of SrAl_2_O_4_ can also be seen for SiO_2_@SAOED and NH_2_@SiO_2_@SAOED phosphors, indicating that the main structure of the SAOED phosphors was not affected by SiO_2_ and APTMS modification. The absorption band near 3430 cm^−1^ in the spectra of SiO_2_@SAOED and NH_2_@SiO_2_@SAOED phosphors is assigned to the symmetric stretching of their hydrated hydroxyl groups [35]. For the SiO_2_@SAOED phosphors, a strong absorption peak corresponding to the asymmetric stretching of Si-O-Si groups is observed at 1079 cm^−1^, indicating that a SiO_2_ layer was successfully coated on the surface of SAOED particles. In the case of NH_2_@SiO_2_@SAOED phosphors, the Si–O–Si stretching vibrations are observed at 1087 and 1050 cm^−1^, the N–H stretching vibration (about 3417 cm^−1^) is not distinguished due to its overlap with the wide absorption band of –OH at 3430 cm^−1^ [36]. However, an additional absorption peak corresponding to the stretching of –C–H group is observed at 2926 cm^−1^ [36]. These results suggest that APTMS might react with the hydroxyl groups of the SiO_2_@SAOED particles, resulting in grafting –NH_2_ groups onto the surface of phosphors.

In order to demonstrate the importance of pre-modification of SiO_2_, uncoated phosphors (SAOED) and pure APTMS modified particles (NH_2_@SAOED) were immersed in water for 10 to 360 min, respectively. The FTIR spectra (Figure 2b) show that the main characteristic peak of phosphor particles at 850 cm^−1^ disappeared gradually due to their severe hydrolysis without SiO_2_ pre-coated modification. These results are in accordance with the pH values of phosphor aqueous suspensions in Figure 2c. For SiO_2_@SAOED and NH_2_@SiO_2_@SAOED phosphors, the pH of the suspensions are basically stable for more than 8 h due to the good water resistance of SiO_2_ preformed coating layers on SAOED phosphors. The successful grafting of SiO_2_ and APTMS onto the phosphors to form a core-shell structure was further confirmed by the XPS results (Figure 2d). The XPS results clearly show the binding energies of O (1s, 532.8 eV) and Si (2s, 154.6 eV; 2p, 103.4 eV) for SiO_2_@SAOED and that of N (1s, 401.6 eV) and C (1s, 284.8 eV) for NH_2_@SiO_2_@SAOED phosphors. Furthermore, the reversal Zeta potential values of modified phosphors (Figure 2f), being transferred from –25.3 to +19.6 mV after amino-modification, also proved that APTMS had grafted onto SiO_2_-coated phosphors. As shown in Figure 2e, the results of XRD analysis for SiO_2_@SAOED and NH_2_@SiO_2_@SAOED showed that no new characteristic peaks except a broad band (2θ = 20°–25°) for amorphous SiO_2_ (JCPDS 29-0085) compared with the characteristic crystal plane index on (011), (211), (220), (211), and (031) for uncoated SrAl_2_O_4_ sample (JCPDS 74-0794). This indicates that the SiO_2_ coating crystallized well onto the surface of SAOED phosphors. This result is consistent with that of SiO_2_-coated SrSO_4_: Sm^3+^ phosphors [37]. Furthermore, for the NH_2_@SiO_2_@SAOED particles, no second phase was detected except for the broad band for amorphous SiO_2_ at 2θ = 22°, indicating that the crystal structure of SAOED particles did not change by APTMS modification.

**Figure 3:**
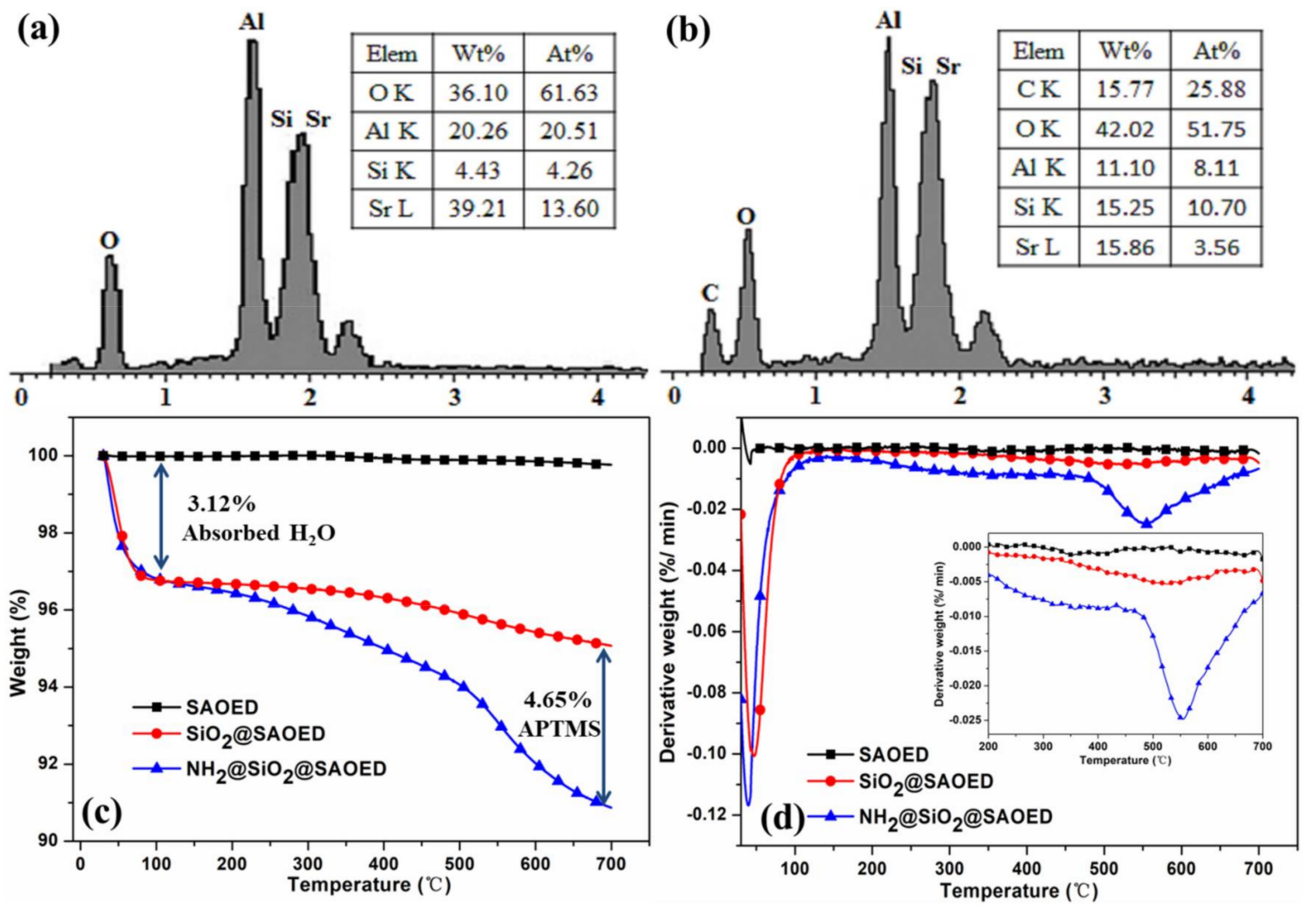
Quantitative analysis of silica and amino-silane on the surface of luminescent particles: energy dispersive analysis (EDS) of (**a**) SiO_2_@SAOED, and (**b**) NH_2_@SiO_2_@SAOED particles; (**c**) Thermogravimetry (TG) and (**d**) Differential thermal gravimetric (DTG) analysis curves of SAOED, SiO_2_@SAOED, and NH_2_@SiO_2_@SAOED.

EDS was carried out to analyze the surface components of the coated phosphors. Figure 3a shows the energy dispersive peak of Si at approximately 1.9 eV which could not be distinguished because of the Sr peak, which is consistent with that of reported literature [27]. A detailed analysis of the EDS results showed that the relative content of Si distributed on the surface of luminescent particles was about 4.43%. The EDS profile of NH_2_@SiO_2_@SAOED (Figure 3b) showed an additional signal corresponding to C apart from the signals from SiO_2_@SAOED, which indicates that APTMS was successfully coated on the SiO_2_@SAOED particles. Considering that the APTMS contains three methylene (–CH_2_) groups and the relative C content was 15.77%, it was inferred that the amount of APTMS coated onto the phosphor surface was 5.26%.

The formation of APTMS coating on SiO_2_@SAOED was further confirmed by the thermal decomposition behavior using TGA. Figure 3c,d show the thermogravimetry (TG) and differential thermal gravimetric (DTG) analysis curves of SAOED and modified phosphors. As shown in Figure 3c, the mass changing rate of uncoated SAOED phosphors basically remained constant at 700 °C. It was mainly because the phosphors were prepared through a high temperature sintering method in a reducing hydrogen airflow or using activated carbon [38,39]. On the other hand, the coated particles (SiO_2_– and SiO_2_@APTMS-coated) showed a rapid weight loss of 3.12% at low temperatures (≤120 °C) because of the removal of adsorbed water. The DTG curve for NH_2_@SiO_2_@SAOED showed a sharp peak after 450 °C, which was attributed to the weight loss caused by the thermal decomposition of the amino-silane molecules that chemically bonded on the phosphors [40]. The weight loss at 700 °C for SiO_2_@SAOED and NH_2_@SiO_2_@SAOED was calculated to be 4.56% and 9.21%, respectively. Therefore, the amount of APTMS deposited on SiO_2_-coated particles was estimated to be about 4.65%, which is basically consistent with the EDS analysis results. Combined with TEM, FTIR, XPS, and EDS analyses, all of these results gave evidence that SiO_2_ and APTMS had successfully coated onto SAOED phosphors, respectively.

**Figure 4:**
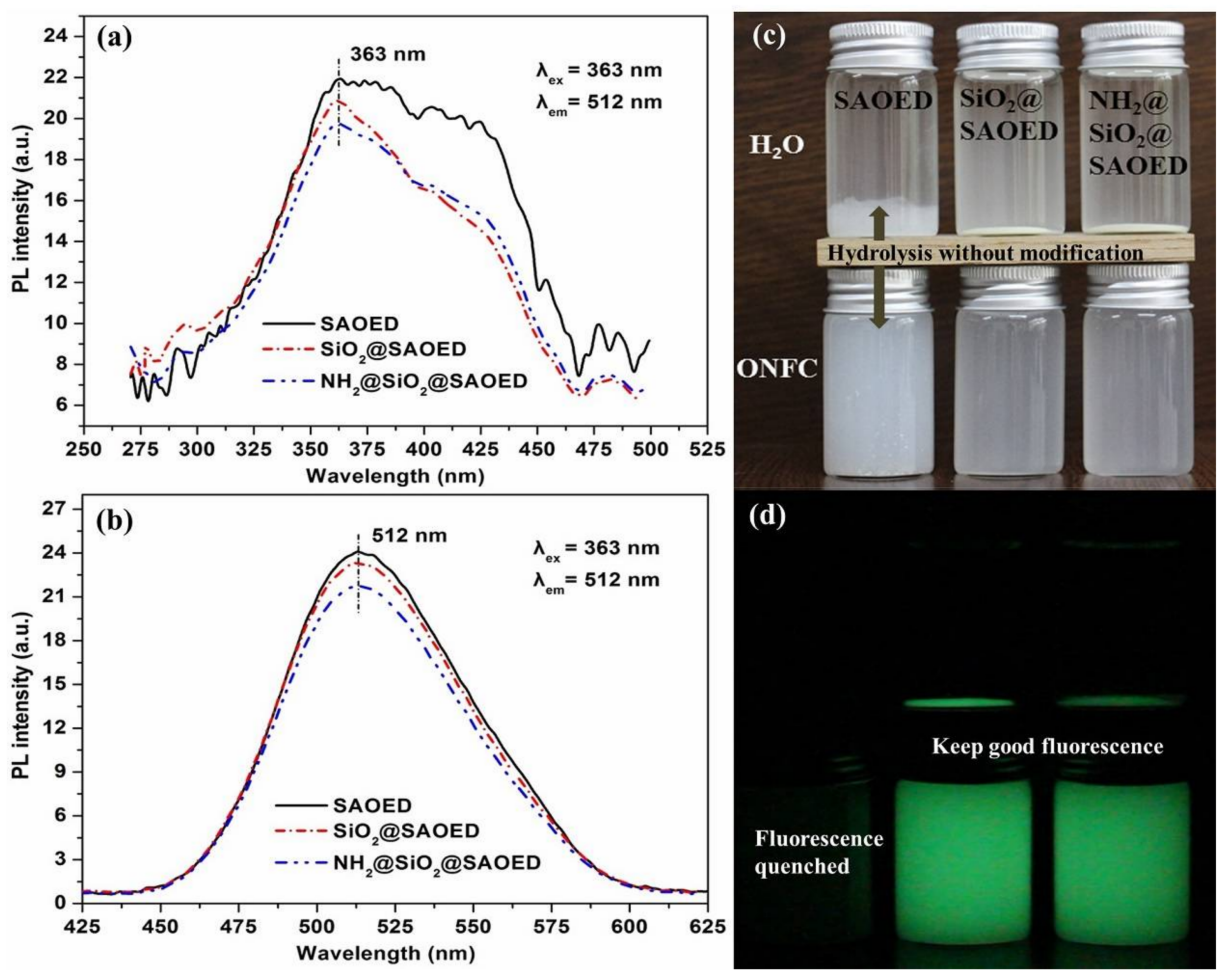
Photoluminescent (PL) spectra of SAOED phosphors and different suspensions in water and ONFC: (**a**) excitation and (**b**) emission spectra of phosphors before and after surface coated modification. Excitation wavelength λ_ex_ = 363 nm was considered for emission spectra measurements; Digital photographs of the phosphors dispersed into distilled water (first row) and ONFC suspension (second row) for 24 h (**c**) in daylight and (**d**) in darkness.

The excitation spectra of the phosphors (Figure 4a) show a continuous wide band centered at 363 nm, corresponding to the typical 4f^7^-transition of Eu^2+^ ions, indicating that the SAOED phosphors were effectively excited by the ultraviolet to visible light [40]. After modification, the region near excitation peak narrowed while the peak was still at 363 nm. Figure 4b shows the emission spectra of the SAOED samples. It can be clearly seen that all the samples exhibited a single emission peak at 512 nm under 363 nm of excitation, which is assigned to the 4f^6^5d^1^ to 4f^7^ transition of Eu^2+^ ions [41,42]. The intensity of emission spectra was 3.46% and 9.71% lower for SiO_2_@SAOED and NH_2_@SiO_2_@SAOED, respectively compared to that of the SAOED sample. It can be attributed to the light scattering and reflection due to the SiO_2_ and APTMS coating layers on the outer surface of the phosphors. These results are in good agreement with the study which found that a macromolecule network structure might reduce the excitation and emission peak of phosphors [22]. Therefore, the spectra of SAOED phosphors after modification did not significantly change, indicating that both the coating methods were suitable for modification of the SAOED phosphors.

### 3.2. Preparation of Suspension and Luminescent Composite Films

**Figure 5:**
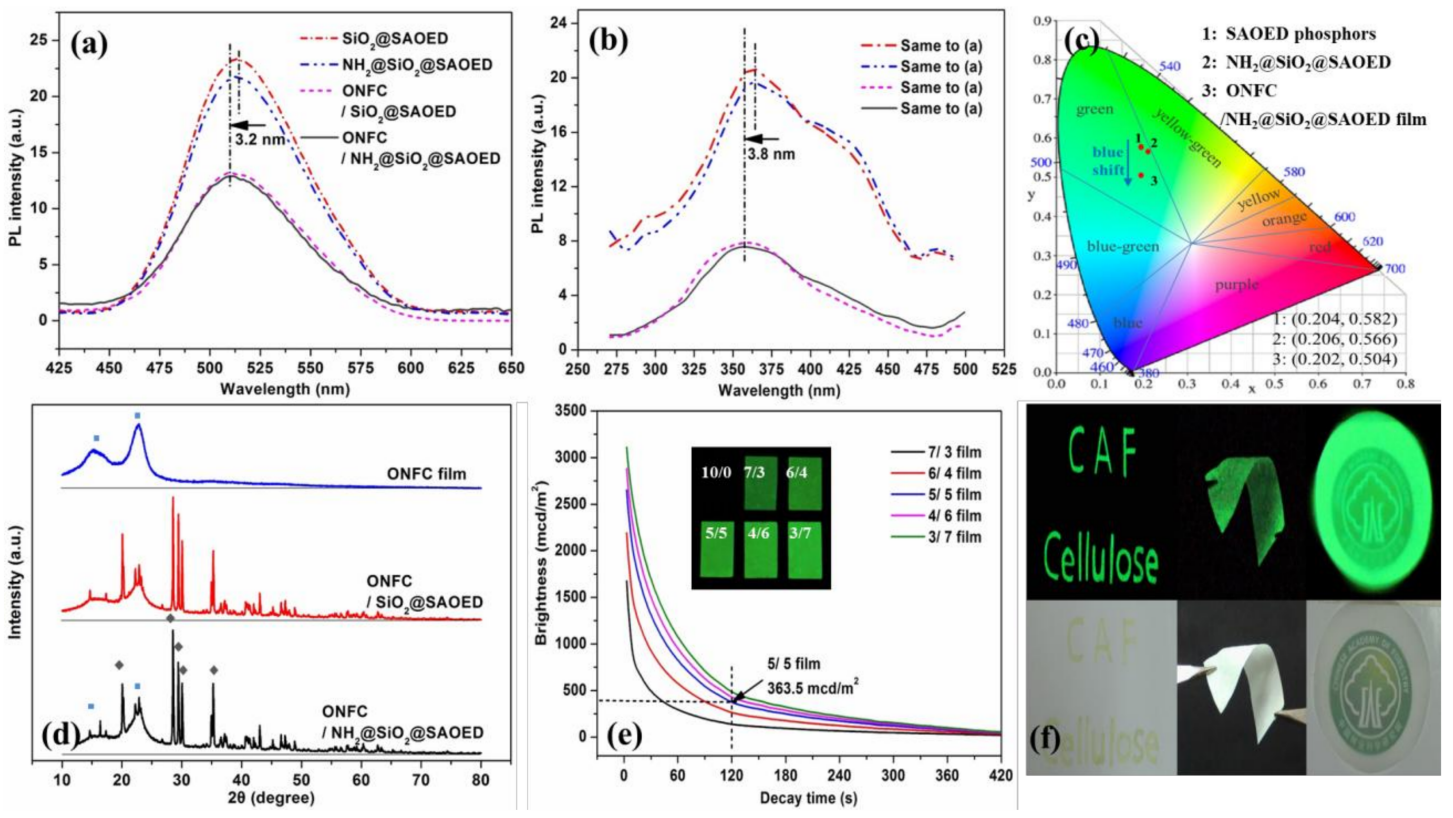
Photoluminescent (PL) performance for the SAOED phosphors and ONFC luminescent films: (**a**) emission and (**b**) excitation spectra of ONFC luminescent films with modified phosphors (ONFC/phosphor = 1/1); (**c**) CIE chromaticity diagram of SAOED phosphors before and after modification; and ONFC luminescent films with NH_2_@SiO_2_@SAOED phosphors in 1/1 mass ratio; (**d**) X-ray diffraction patterns for ONFC film, ONFC luminescent film with SiO_2_@SAOED and NH_2_@SiO_2_@SAOED phosphors (mass ratio = 1/1); (**e**) Decay curve of films with NH_2_@SiO_2_@SAOED in different mass ratios (inset: digital pictures of films in 2 min under dark); (**f**) Some potential examples of luminescent logo or flexible labels in darkness (first row) and daylight (second row).

To prepare flexible luminescent films, the ONFC suspensions with modified phosphors were homogeneously blended. Figure 4c,d show the photographs of the SAOED, SiO_2_@SAOED, and NH_2_@SiO_2_@SAOED samples dispersed in water and ONFC suspensions. Both the modified and uncoated phosphors appeared to be sinking in water. The mixture obviously had two phases with most of the phosphors deposited at the bottom of the bottles. However, phosphors after modification still maintained luminescence in water. As confirmed from the SEM and FTIR results, the SiO_2_ layer on the SAOED particles prevented the H_2_O molecules from entering the SrAl_2_O_4_ channel and enhanced their water resistance. On the other hand, all the samples were miscible in the ONFC suspension and no phase separation was observed. However, the uncoated phosphors did not exhibit any afterglow luminescence in the dark compared with the modified particles, which can be attributed to the formation of alumina floccus because of the hydrolysis of SrAl_2_O_4_ in water [18]. While the modified particles formed uniform suspensions in the ONFC matrix this was mainly attributed to the formation of hydrogen bonds between the nanocellulose and the SiO_2_@SAOED particles and additional electrostatic adsorptions for the NH_2_@SiO_2_@SAOED and nanocellulose by an opposite charge [31,32]. The presence of a sufficient amount of nanocellulose and their small size ensured the stability of the mixed suspensions. The homogeneous suspensions with luminescence were necessary for the fabrication of films. This was confirmed by the XRD pattern (Figure 5d) of ONFC luminescent films, showing the diffraction peaks for both the crystalline ONFC and SrAl_2_O_4_.

### 3.3. Characterization of Luminescent Films with Modified Phosphors

Figure 5a,b show the photoluminescent (PL) spectra of the ONFC composite films with different modified phosphors (ONFC/phosphor = 1/1). The PL luminescent properties of the films were consistent with that of the coated phosphors. However, it was found that the emission spectra of the composite films showed a slight blue shift (3.2 nm) at around 512 nm compared to modified phosphors. This was probably due to the variations in the local microenvironment including the nano-sized effect of nanofibrillated cellulose and the introduction of moisture during the preparation of films. Despite the occurrence of a slight blue shift for the luminescent films [43,44,45], the luminous color still belongs to green (Figure 5c). The XRD pattern of ONFC luminescent films (Figure 5d) shows the diffraction peaks for both the crystalline ONFC (2θ = 16.5° and 22.5°) and SrAl_2_O_4_, which further confirmed that the SiO_2_ and SiO_2_@APTMS modification effectively restrains the hydrolysis of SAOED phosphors. Figure 5e presents the PL decay curves of films with amino-modified phosphors in different mass ratios. All the luminescent films showed a rapid decay during the first 2 min and a slow decay process subsequently. Even though the initial afterglow brightness of films increased gradually with an increase in the proportion of phosphors, the increase was not obvious at higher phosphor contents, especially for the films with the phosphor ratios of more than 1/1. The brightness of films with the ONFC/phosphor ratios of 5/5, 4/6, and 3/7 after 2 min was 363.47, 417.25, and 471.04 mcd/m^2^, respectively. This can be ascribed to the varying depths of the trap levels in the films [28]. Additionally, only the control film showed no afterglow luminescence under the same test conditions. As shown in Figure 5f, some photoluminescent labels were prepared to exhibit the potential applications of this functional film.

**Figure 6:**
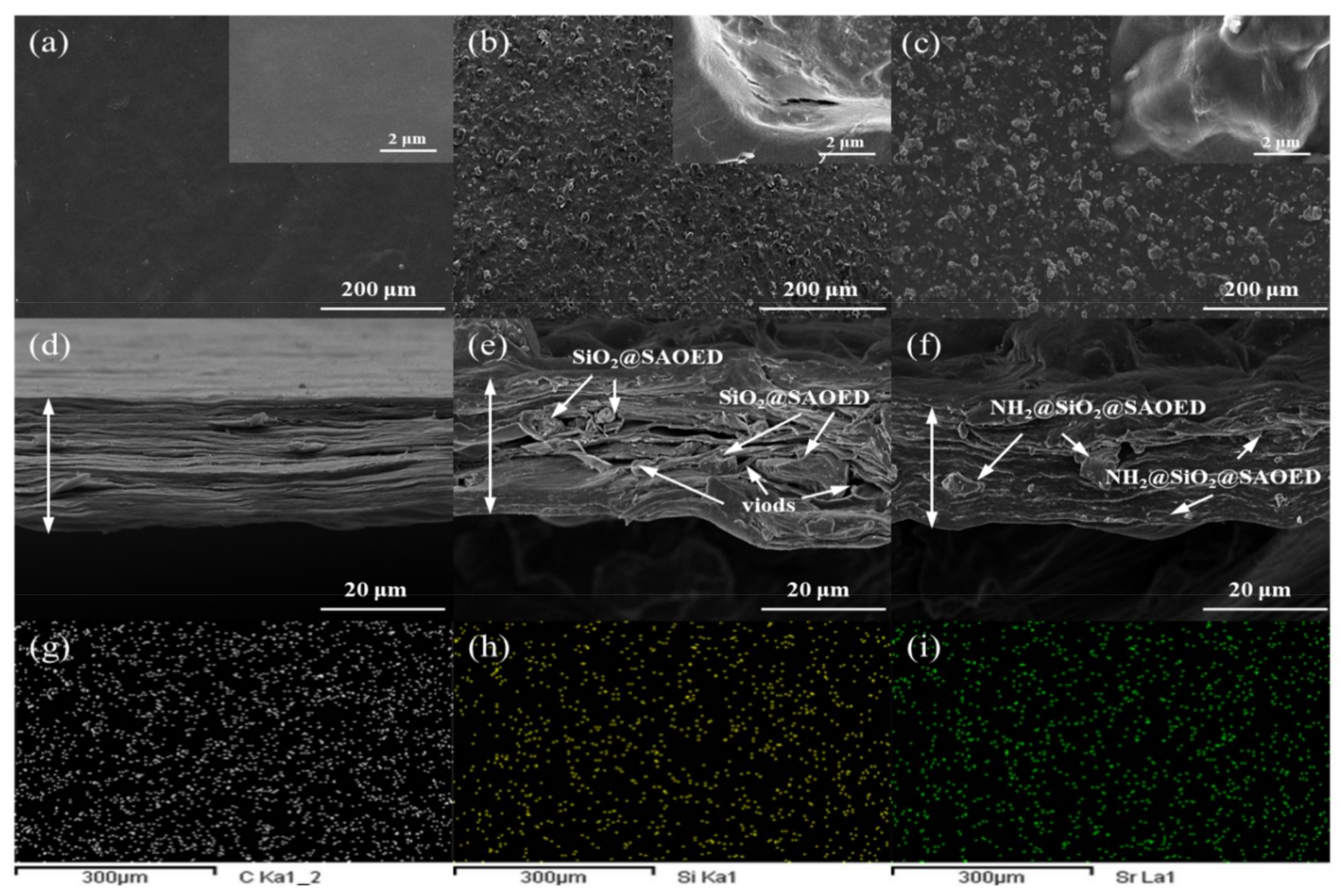
Typical micromorphology and particle dispersion of ONFC luminescent films: SEM morphologies of the surface and the cross-section of (**a**,**d**) ONFC film (inset: high magnification image of composite film); (**b**,**e**) ONFC luminescent film with SiO_2_@SAOED (mass ratio = 1/1); and (**c**,**f**) ONFC luminescent film with NH_2_@SiO_2_@SAOED phosphors (mass ratio = 1/1); EDS-mapping patterns of luminescent film with NH_2_@SiO_2_@SAOED phosphors after scanning the characteristic elements of (**g**) carbon; (**h**) silicon; and (**i**) strontium.

Figure 6 shows the typical SEM morphologies of the control ONFC film, and ONFC luminescent films with modified phosphors (mass ratio = 1/1). In Figure 6b,c, it is shown that the microstructure of cellulose nanofibers was closely stacked when the homogeneous ONFC suspensions were dehydrated. Compared to the smooth surface of control ONFC film in Figure 6a, the modified particles were uniformly dispersed in the ONFC matrix. These results are consistent with the blending results shown in Figure 4c,d and Figure 6g–i. From Figure 6e,f, it can be observed that the modified phosphors were wrapped by the nanofibrils and were uniformly fixed in the skeleton of fiber cross-linked structure. However, compared to the SiO_2_@SAOED film, the film containing NH_2_@SiO_2_@SAOED showed a more closely packed structure and had less voids at the interface between the cellulose and luminescent particles. This can be attributed to the poor activity of micron-sized silica particles. On the other hand, the amino-silanized particles with a positive charge (+19.6 mV) were expected to yield electrostatic adsorption [32] during the process of blending with ONFC.

**Figure 7:**
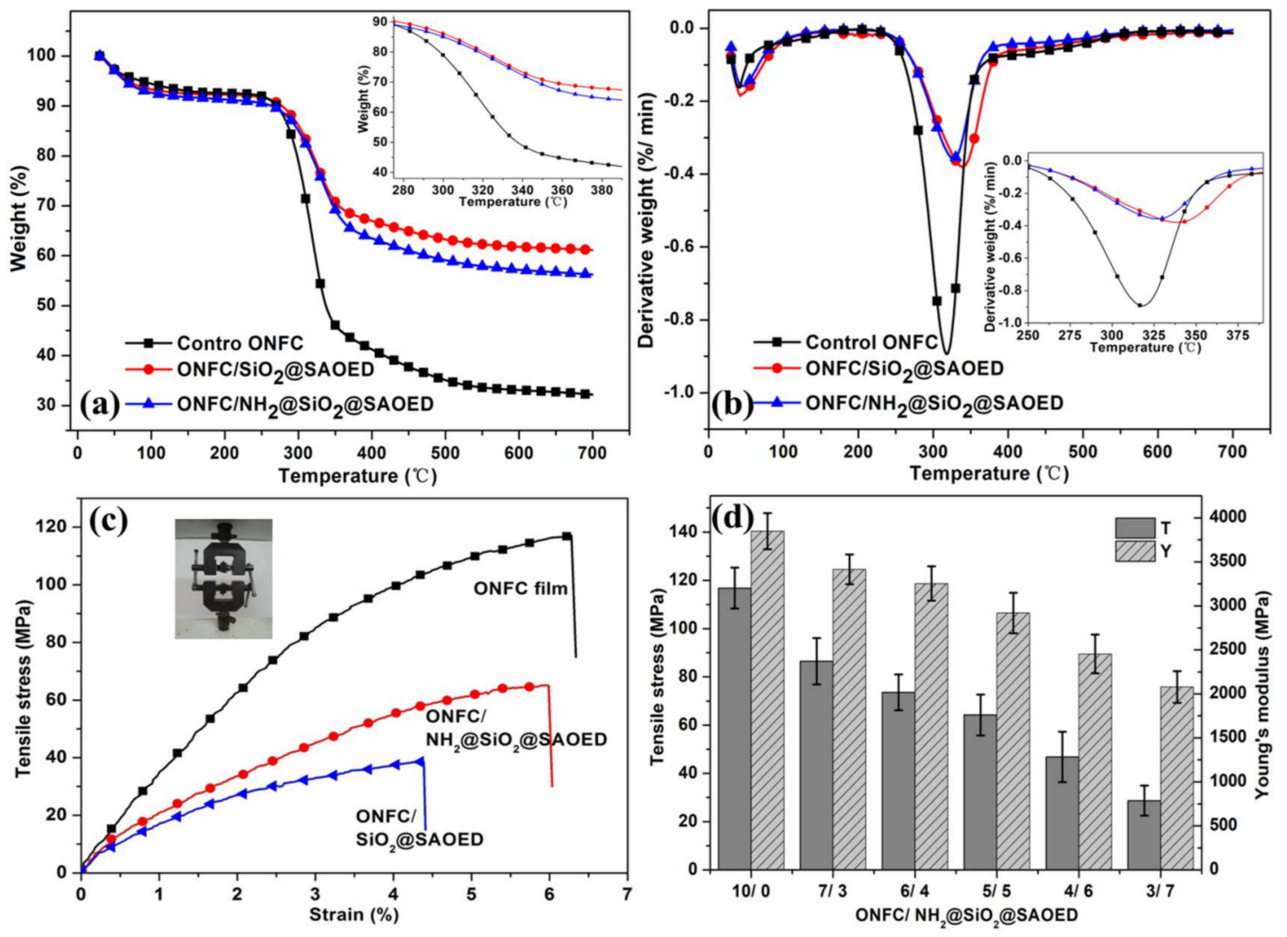
Thermal analysis and tensile strength of the ONFC luminescent films: (**a**) TG and (**b**) DTG curves of luminescent composite films with different modified SAOED phosphors (mass ratio = 1/1) compared with control ONFC film; (**c**) Stress-strain curves of tensile test results of control ONFC film, composite films with different modified SAOED phosphors (mass ratio = 1/1); (**d**) Tensile strength of ONFC/NH_2_@SiO_2_@SAOED luminescent films with different mass ratios.

The TG and DTG curves of the ONFC composite films are shown in Figure 7a,b. The thermal stability of luminescent films with modified phosphors showed significant improvement in comparison to the control ONFC film. The TG curves revealed that the films had two stages of rapid degradation. The initial thermal degradation was observed in 265 to 345 °C. However, the thermal decomposition rate of the films with NH_2_@SiO_2_@SAOED phosphors was a little higher than that of the films with SiO_2_@SAOED particles. This is consistent with the results of modified phosphors (Figure 3). For the luminescent films, the second decomposition peak showed a slight shift toward higher temperatures, as shown in Figure 7b. This can be attributed to the addition of phosphors, which restrained the heat transfer process. Furthermore, the char yield of the luminescent films was much higher than that of the control film at 265 to 700 °C (Figure 7a). The reason is the good thermal stability of the phosphors, which is also observed from Figure 3c. The char residue of the films with SiO_2_@SAOED and NH_2_@SiO_2_@SAOED phosphors at 700 °C was 61.35 and 57.14 wt %, respectively, while that of the control film was 32.48 wt %. This is consistent with the thermal degradation behavior of the modified phosphors.

The stress-strain curves of the films with different modified phosphors (mass ratio = 1/1) are shown in Figure 7c. Figure 7d shows the average tensile strength and Young’s modulus of the ONFC/NH_2_@SiO_2_@SAOED films with different mass ratios. As shown in Figure 7c, the tensile strength of the films with phosphors was lower than that of the native ONFC film. It was mainly because the incorporation of phosphors into the ONFC network reduced the number and strength of the hydrogen bonds for the nanocellulose. This resulted in the accumulation of stress at the interface between the modified phosphors and the cellulose. However, the mechanical strength of the film with NH_2_@SiO_2_@SAOED particles was much higher than that of the film with SiO_2_@SAOED particles. The reason behind this is the fact that the amino groups (–NH_3_^+^) on the surface of aminated phosphors tend to produce electrostatic attraction with the cellulose carboxylate groups (–COO^−^), which enables a strong interaction between the modified phosphor and nanofibrillated cellulose while the hydrogen bonds between cellulose and SiO_2_@SAOED phosphors were not strong enough to produce sufficient interaction with inorganic micro-particles. The tensile strength (T) and Young’s modulus (Y) (Figure 7d) show that the 3/7 composite films had the lowest T and Y values. This can be attributed to the gradual weakening of the cellulose network with an increase in the phosphor amount. Similar results have been reported for other ONFC films [31]. The tensile stress of the 1/1 (ONFC/phosphor) composite film was 64.2 MPa, and the Young’s modulus decreased only by 24.16% from 3850 to 2920 MPa. This indicates that the incorporation of amino-modified SAOED phosphors into the ONFC matrix at a mass ratio of 1/1 produces luminescent films with good tensile properties.

## 4. Conclusions

Surface modified SrAl_2_O_4_: Eu^2+^, Dy^3+^ phosphors (using TEOS and APTMS) were successfully introduced into the TEMPO-oxidized nanofibrillated cellulose (ONFC) matrix using an environment-friendly template method to manufacture high-strength flexible films with long afterglow luminescence. Nano-scaled SiO_2_ and APTMS coatings were formed on the surface of the SAOED particles after the modification. The incorporation of the SiO_2_@SAOED or NH_2_@SiO_2_@SAOED phosphors into the ONFC matrix resulted in the formation of stable and homogeneous suspensions without destroying the crystal structure. The emission spectra of composite films showed a slight blue shift at around 512 nm. The afterglow and tensile tests showed that the composite films with NH_2_@SiO_2_@SAOED phosphors exhibited good luminescence and better mechanical properties compared to that of film with silica-modified particles. The results obtained from this study suggest that the incorporation of SiO_2_@APTMS-aminated phosphors into TEMPO-oxidized nanofibrillated cellulose (ONFC) matrix is a feasible, green, and effective method for the fabrication of luminescent films for applications such as luminous flexible equipment, night indication, and portable logo or labels.

## Supplementary Materials

The following are available online at http://www.mdpi.com/2079-4991/8/5/352/s1.
