# Supplementary material for: Fabrication Flexible and Luminescent Nanofibrillated Cellulose Films with Modified SrAl2O4: Eu, Dy Phosphors via Nanoscale Silica and Aminosilane
Source: Nanomaterials (Basel). 2018 May 22;8(5):352. doi: 10.3390/nano8050352 (PMC5977366; doi:10.3390/nano8050352)

Supporting Information

Figure S1. SEM micrographs of $SrAl_2O_4$: Eu^{2+}, Dy^{3+} (SAOED) phosphors before and after coated modification: (a) native SAOED phosphors; (b) SiO_2 coated SAOED (SiO_2@SAOED); (c) APTMS coated SiO_2@SAOED phosphors (NH_2@SiO_2@SAOED).

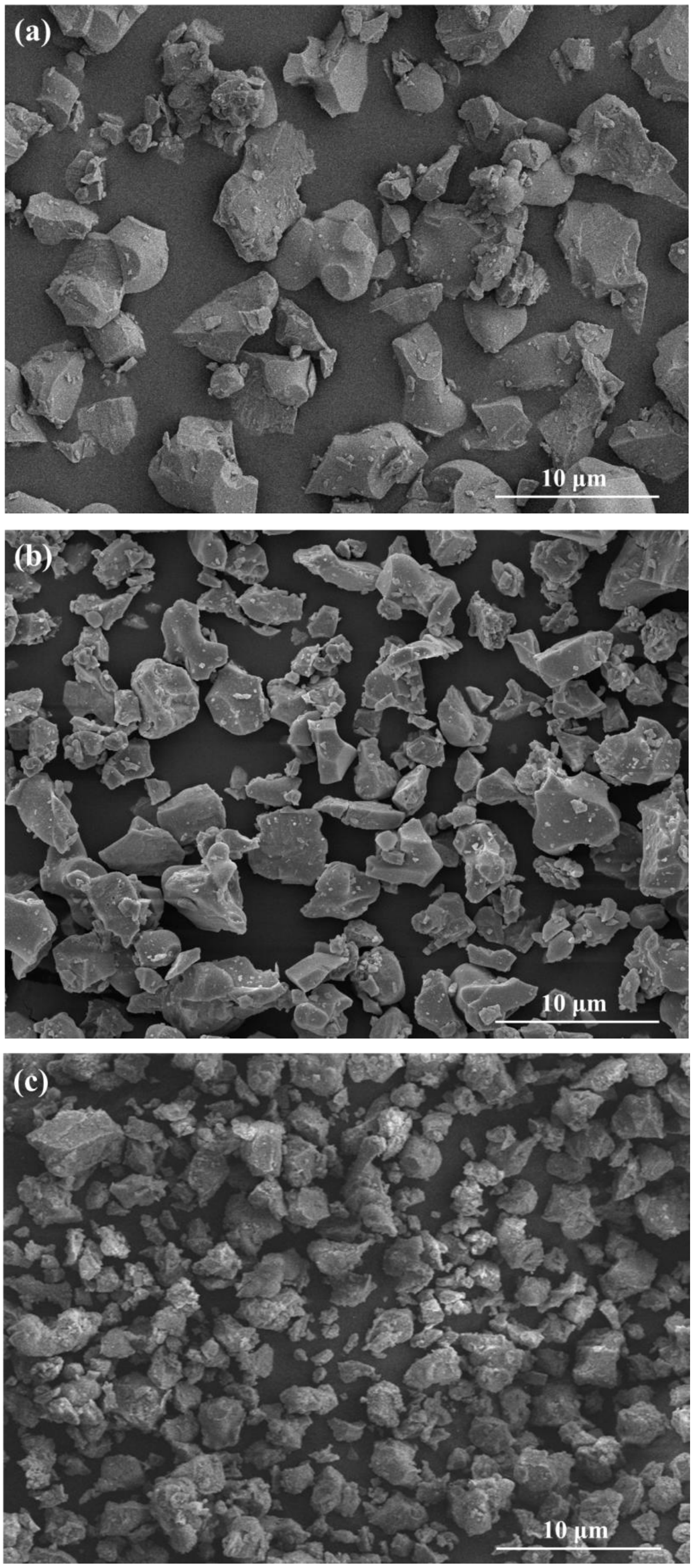

Supplement: Supplementary file 1 [file nanomaterials-08-00352-s001.pdf]
